# QTL analysis of four main stem bark traits using a GBS-SNP-based high-density genetic map in ramie

**DOI:** 10.1038/s41598-017-13762-w

**Published:** 2017-10-18

**Authors:** Chan Liu, Siyuan Zhu, Shouwei Tang, Hongwu Wang, Xia Zheng, Xiaorong Chen, Qiuzhong Dai, Touming Liu

**Affiliations:** 1 Institute of Bast Fiber Crops, Chinese Academy of Agricultural Sciences, Changsha, China; 2 Xianning Agriculture Academy of sciences, Hubei, China; 3 Yichun Institute of Agricultural Sciences, Jiangxi, China

## Abstract

Ramie fiber extracted from ramie stem bark (RSB) is a highly important natural fiber, and therefore, RSB is an economically important plant organ. The genetic basis of RSB traits is poorly understood. In the present study, fiber yield and three RSB traits (bark thickness, bark weight, and fiber output ratio) were subject to quantitative trait locus (QTL) analysis using an F_2_ agamous line population derived from two ramie varieties (Qingdaye and Zhongzhu 1). A total of 4338 high-quality single nucleotide polymorphisms were identified using the genotyping-by-sequencing technique and were subsequently used to construct a high-density genetic map spanning 1942.9 cM. Thereafter, QTL analysis identified five, two, four, and four QTLs for bark thickness, bark weight, fiber output ratio, and fiber yield, respectively. A 5.1 cM region that corresponded to a QTL for bark thickness (*qBT4a*) contained 106 candidate genes, and the Zhongzhu 1 allele of one of the genes, a putative MYB gene (*evm.model.scaffold7373.133_D1*), included a 760-bp insertion that caused premature termination, thereby producing a protein that lacked part of the MYB domain. Because MYB transcription factors play central roles in regulating the development of secondary cellular walls and fiber biosynthesis, we propose *evm.model.scaffold7373.133_D1* as a likely candidate gene for *qBT4a*.

## Introduction

Ramie (*Boehmeria nivea* L. Gaud), which is commonly known as China grass, is a perennial and diploid (2n = 28) herbaceous plant that belongs to the Urticaceae and is one of the most important natural fiber crops. Ramie fibers are vegetative and are extracted from the plant’s stem bark. Therefore, fiber yield (FY) is markedly influenced by ramie stem bark (RSB) traits, like bark thickness (BT), bark weight (BW), and the output ratio of fiber from the bark (RFB), and understanding the genetic basis of these traits will facilitate molecular designing of the traits, as well as breeding programs for FY.

Many of ramie agronomic traits, including FY and RSB traits, are complex and are inherited in a quantitative manner^1,2^, which suggests that the traits are controlled by a variety of major and minor quantitative trait loci (QTLs). Owing to the development of molecular marker and QTL analysis methods during the past 20 years, a great number of quantitative traits have been characterized for important crop species, and thousands of corresponding QTLs have been identified^2–8^, some of which have also been fine-mapped and cloned^9–13^. In recent years, numerous SSR markers have also been developed in ramie, based on expressed sequence tags (ESTs) from the *de novo* assembled transcriptome of species^14,15^. However, only one expanded, low-resolution genetic map has been constructed^2^, and only 33 and 29 QTLs have been detected for yield- and flowering time-related traits, respectively^2,8^. Therefore, it is likely that many QTLs have yet to be identified, because of the QTL regions without markers observed in this low-resolution map, and a high-resolution genetic map of ramie is needed.

Ramie breeding programs often focus on RSB-related traits, owing to their effects on FY. However, only BT was included in the previous analysis that only identified six QTLs^2^, and no RSB development-related genes or QTLs have been cloned or functionally characterized. Therefore, the genetic and molecular mechanisms for the RSB traits remain completely unknown.

A major objective of this study is to characterize the genetic basis of RFB traits. To achieve this aim, whole-genome single nucleotide polymorphism (SNP) was identified, and used to construct a high-density genetic linkage map of ramie, using a genotyping-by-sequencing (GBS) technique and an F_2_ agamous line (FAL) population that consisted of 134 lines derived from two ramie varieties, Qingdaye (QDY) and Zhongzhu 1 (ZZ1). Thereafter, QTL analysis for FY and three RSB traits was performed; and based on the QTL region sequences of two the parents, candidate gene for one of the BT QTLs (*qBT4a*) was further investigated. The present study provides a basis for understanding the genetic and molecular mechanisms of RSB development and will facilitate future breeding programs improving ramie fiber yield.

## Results

### GBS-based SNP identification

Over 329 million clean 125-bp reads were generated from the libraries of two parents and 134 F_2_ agamous lines (FALs) and were deposited in the NCBI-short read archive (SRA) database under accession number SRX2830696. Most of the sequence reads were uniquely aligned to the ramie genome, with an average mapping rate of 85.5% among 136 families (Table S1). From these reads, 489,057 sequence tags were generated, with depths of 9.11- to 17.26-fold (Table S1), and the sequence tags that aligned uniquely to the genome were used for genome-wide high-throughput SNP identification. A total of 197,353 unfiltered SNPs between two parents, QDY and ZZ1, were identified, and 34,870 exhibited the ‘aa × bb’ segregation pattern and were filtered further (Fig. S1), finally yielding 4338 high-quality SNPs.

### High-density genetic map development

**Figure 1:**
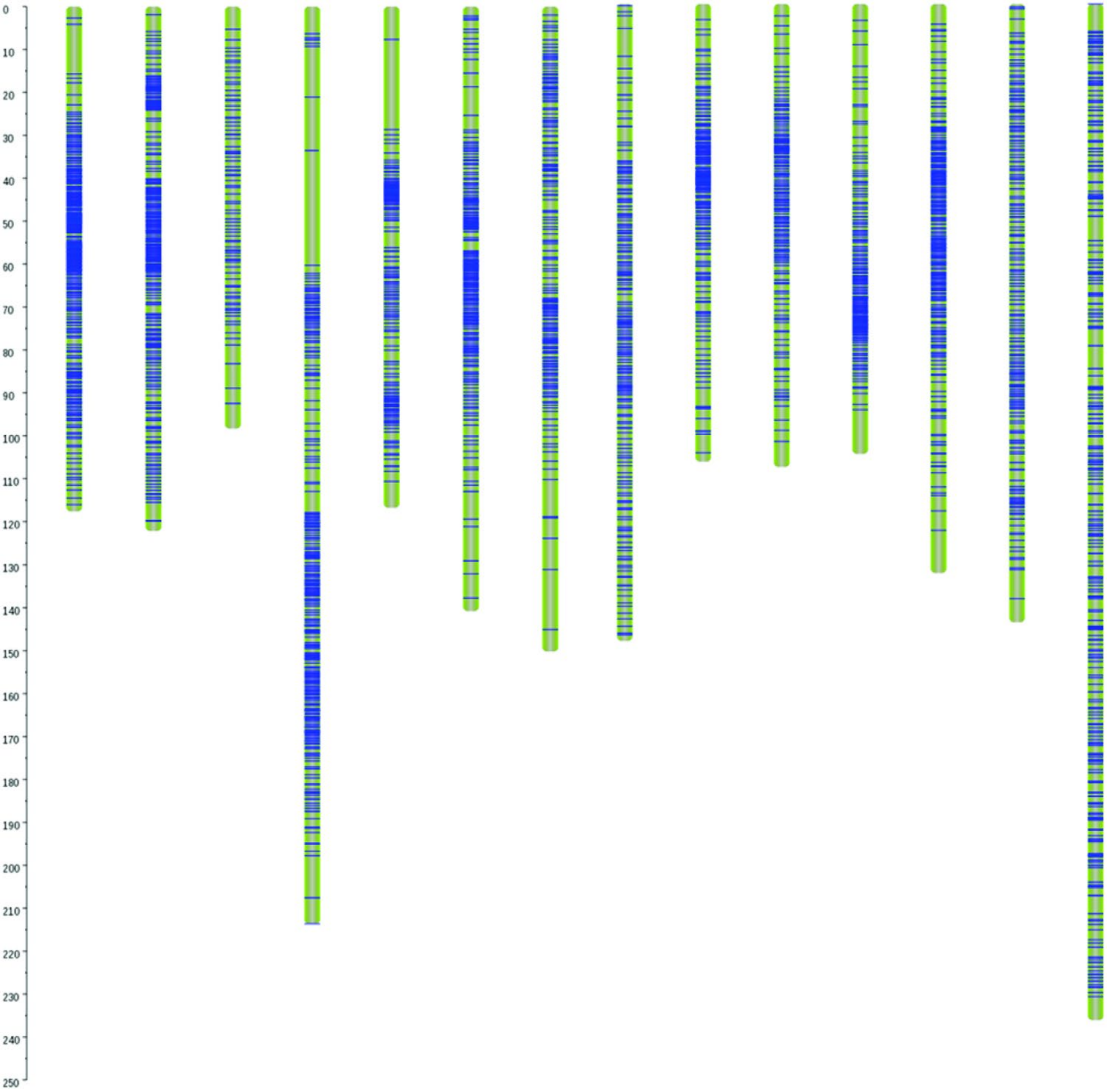
Genotyping-by-sequencing-based high-density genetic map of Qingdaye and Zhongzhu 1 ramie. The 1942.9-cM map includes 4338 SNPs.

The 4338 high-quality SNPs formed 14 linkage groups, with total a length of 1942.9 cM and 2965 SNPs being used as bin markers, and constituted the first high-density genetic map of ramie (Table S2, Fig. 1). The individual linkage groups ranged from 98.3 to 237.3 cM in length, and the SNPs were evenly distributed in the genetic map, with 86–332 binned SNPs per linkage group, an average distance of 0.66 cM, and a maximum distance of 25.2 cM (Table S2). There were only 13 intervals with genetic distances of >10 cM (Table S2).

### QTL mapping of FY and RSB traits

The FY and RSB traits of the two parent varieties (ZZ1 and QDY) were significantly different (Table 1). For example, ZZ1 possessed thick, heavy bark with a high RFB; whereas QDY had thin, light-weight bark and a relatively low RFB. Therefore, the bark from ZZ1 stems yielded more fiber than the bark from QDY stems. During both years, both high variation and transgressive segregation were observed for the four traits in the FAL population (Table 1). The heritability of BT, BW, RFB and FY were 72.8%, 74.3%, 75.6%, and 82.0%, respectively (Table 1). Significant positive correlations were observed among the four traits in the FAL population in the two environments (*P < *0.01), except RFB and BT, and RFB and BW in 2015 (Table 2).

**Table 1:** Descriptive statistics of the traits for the parents and the FAL population.

Traits	Heritability (%)	Year	Range	Mean ± SD	ZZ1	QDY
BT (mm)	72.8	2015	0.38–0.95	0.70 ± 0.10	0.94	0.72
		2016	0.39–0.94	0.58 ± 0.08	0.76	0.60
BW (g)	74.3	2015	5.0–45.3	26.0 ± 8.5	38.3	21.0
		2016	2.4–36.0	16.3 ± 3.9	28.9	17.5
RFB (%)	75.6	2015	8.0–17.7	12.2 ± 2.0	13.34	12.04
		2016	9.8–19.9	14.3 ± 1.8	12.28	10.54
FY (g)	82.0	2015	0.40–6.00	3.17 ± 1.15	5.11	2.53
		2016	0.24–3.87	1.91 ± 0.65	3.55	1.67

**Table 2:** The correlation among BT, BW, FY and RFB.

	BT	BW	FY	RFB
BT		0.68*	0.65*	0.28*
BW	0.81*		0.93*	0.30*
FY	0.68*	0.90*		0.58*
RFB	−0.05	0.07	0.46*	

*Represented the significant correlation at 1%, respectively; the lower and upper numbers of diagonal indicated the correlations among traits in FALs in 2015 and 2016, respectively.

**Figure 2:**
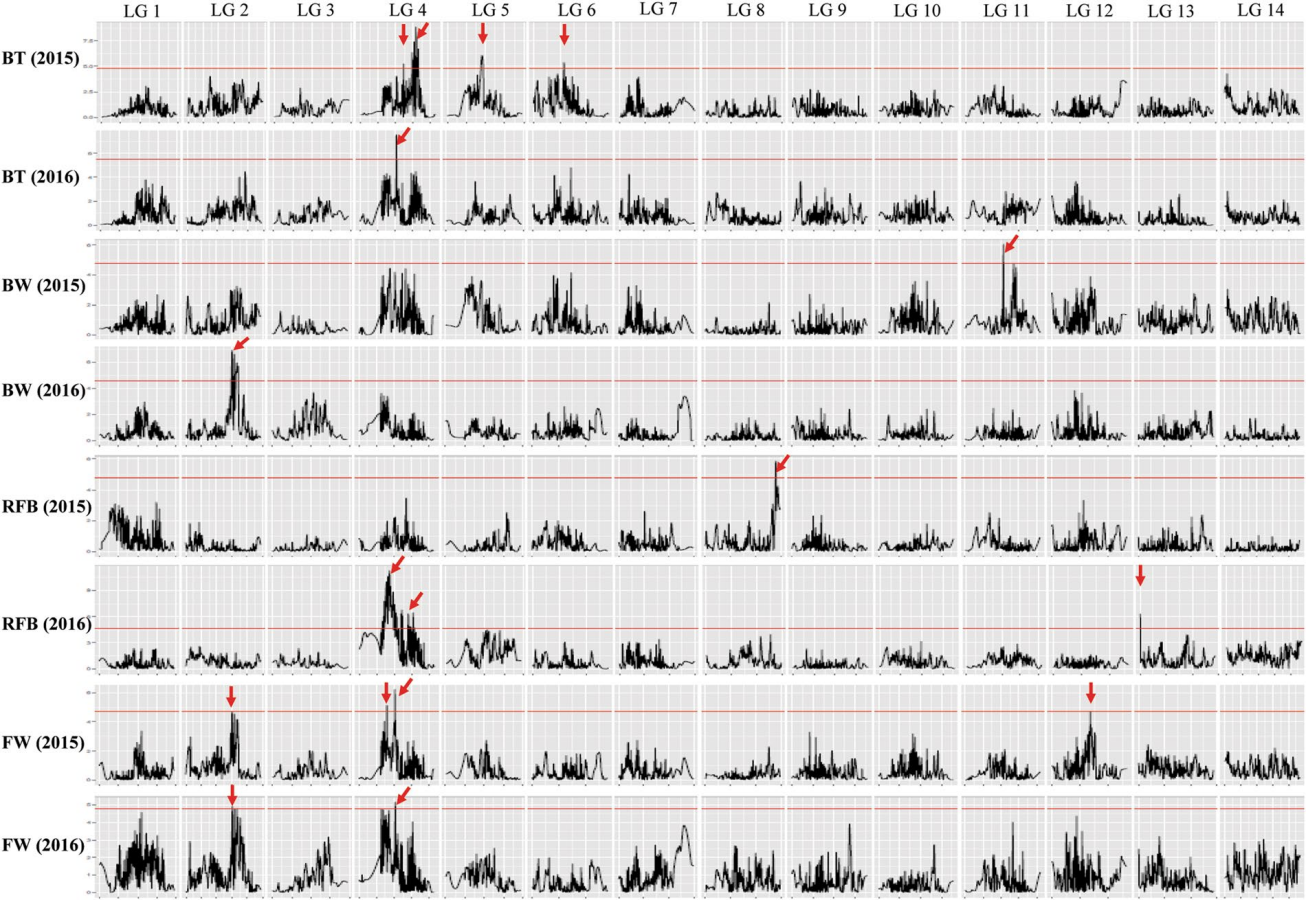
Quantitative trait loci (QTLs) for four ramie stem bark traits. The red line indicates the genome-wide significance LOD threshold, and the red arrow indicates the LOD peak of the QTLs.

The estimated LOD threshold (1000 permutations) indicated that the values for the four traits ranged from 4.6 to 5.5 (Table 3). Based on these LOD threshold values, a total of five, two, four, and four QTLs were identified for BT, BW, RFB, and FY, respectively (Table 4; Fig. 2). Among five BT QTLs, all increased bark thickness by the ZZ1 alleles. For the BW, only one QTL was identified in 2015 and 2016, respectively; among these two BW QTLs, the *qBW11* and *qBW2* increased the weight by ZZ1 and QDY allele, respectively. There were one and three RFB QTLs detected in 2015 and 2016, respectively, and RFB was greater with ZZ1 alleles at the *qRFB4a*, *qRFB4b*, and *qRFB13* loci and the QDY allele at the *qRFB8* locus. In addition, a total of four FY QTLs were identified, two in either year, and yield was improved by the QDY allele at the *qFY2* locus and by the ZZ1 alleles at the other three loci (*qFY4a*, *qFY4b*, and *qFY12*).

**Table 3:** LOD thresholds determined by computing 1,000 permutations (P < 0.05).

Traits	BT	BW	RFB	FY
2015	4.8	4.8	4.8	4.7
2016	5.5	4.6	4.7	4.8

**Table 4:** QTLs identified for four traits from ZZ1/QDY population in two years.

Traits	Year	QTL	LG	LOD value	99%CI (cM)	Add	Var%
BT (mm)	2015	*qBT4a*	4	5.21	122.4–123.0	0.041	8.22
		*qBT4b*	4	8.84	157.7–158.8	0.085	16.28
		*qBT5*	5	6.06	52.0–56.7	0.016	6.61
		*qBT6*	6	5.37	57.2–57.8	0.006	3.79
	2016	*qBT4c*	4	7.53	102.7–105.6	0.092	23.98
BW (g)	2015	*qBW11*	11	6.04	53.0–54.1	6.26	32.78
	2016	*qBW2*	2	6.90	74.3–75.9	−2.21	24.88
RFB (%)	2015	*qRFB8*	8	5.86	138.8–140.3	−1.09	6.77
	2016	*qRFB4a*	4	11.25	85.3–85.7	1.33	12.92
		*qRFB4b*	4	6.08	122.4–123.0	1.40	16.93
		*qRFB13*	13	6.33	0.0–0.7	0.43	7.37
FY (g)	2015	*qFY2*	2	4.70	74.5–76.8	−0.301	8.33
		*qFY4a*	4	5.13	78.6–80.2	0.349	8.60
		*qFY4b*	4	6.24	102.7–104.1	0.352	9.10
		*qFY12*	12	4.73	67.2–68.6	0.580	12.74
	2016	*qFY2*	2	4.91	74.5–76.8	−0.328	9.72
		*qFY4b*	4	5.16	102.7–104.1	0.238	8.281

### Candidate gene prediction for the qBT4a

The *qBT4a* was chosen for further candidate gene analysis. In case of a wave in the QTL location by statistical mapping, an enlarged 5.1-cM region (0.6-cM QTL interval and outer extending regions >2 cM from the bin1448 and bin1337 markers, respectively) that contained 614 kb genome sequence was used to identify the candidate gene of the *qBT4a*. Finally, 106 genes, including genes for a cellulose synthase (CesA; *VIT_07s0005g04110.t01_D1*), a glycosyltransferase (*XP_010091051.1_D2*), a MYB protein (*evm.model.scaffold7373.133_D1*), and a galactosyltransferase (*XP_010091820.1_D1*), were identified (Table S3). Because these proteins have been reported to play important roles in cellulose biosynthesis and secondary cell wall development in model species^16^, the four genes could be related to secondary cell wall development and fiber biosynthesis, thereby influencing BT. Therefore, one of these four genes is likely the candidate gene of the *qBT4a* locus.

**Figure 3:**
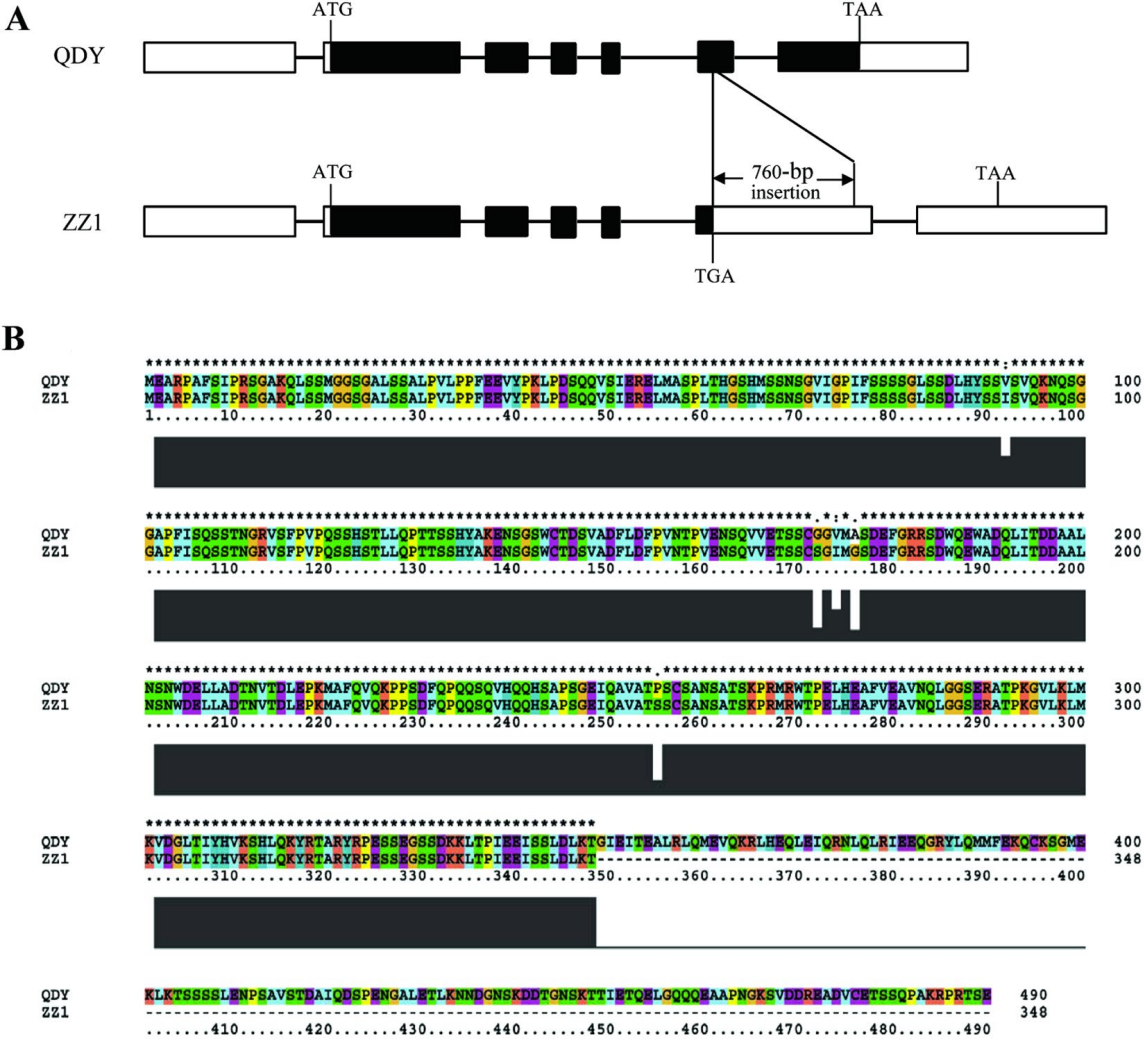
Allele sequences of *evm.model.scaffold7373.133_D1* from Qingdaye and Zhongzhu 1 ramie. (**A**) Zhongzhu 1 allele, including a 760-bp insertion. The rectangle and line represented the exon and intron, respectively, and the black and white rectangles indicate the coding sequence (CDS) and untranslated region (UTR), respectively. (**B**) Clustal X alignment of Qingdaye and Zhongzhu 1 protein sequences.

In order to further investigate the *qBT4a* locus, the cDNA sequences of the four candidate genes were identified in the two parent transcriptomes, which had been assembled previously^17,18^ (Table S4). No key mutations were observed in *VIT_07s0005g04110.t01_D1*, *XP_010091051.1_D2*, or *XP_010091820.1_D1* (Table S4), which suggested that none of these three genes were related to the QTL. However, the ZZ1 allele of *evm.model.scaffold7373.133_D1* included a 760-bp insertion (Fig. 3A), as well as 14 SNPs that caused five amino acid substitutions. These sequence differences were validated using Sanger sequencing. Furthermore, the gene comprises seven exons (Fig. 3A), and the QDY allele encodes a putative MYB transcription factor of 490 amino acids in length (Fig. 3B). In the ZZ1 allele, the 760-bp insertion is found in the sixth exon, and the first codon in the insertion is a termination codon (TGA; Fig. 3A) that results in a 348-amino acid truncation in the C-terminus of the predicted protein (Fig. 3B), thereby eliminating part of the MYB domain (Fig. S2). Because MYB proteins have important roles in regulating cellulose biosynthesis^16^, the truncation of the MYB domain in the putative ZZ1 protein is likely to change its function in fiber biosynthesis. In other words, *evm.model.scaffold7373.133_D1* is a likely candidate gene for the *qBT4a* locus.

To validate our conclusion, cDNA sequence of *evm.model.scaffold7373.133_D1* in four varieties, including two thick-bark (Dazhuhuangbaima and Chuanzhu 8) and two thin-bark varieties (Qingyezhuma and Huazhu 5; Table S5), was extracted from their transcriptomes^19–21^, and was further compared. Although there was not large-fragment insertion observed in four varieties, many nucleotide substitutions in the cDNA sequence were found, resulting in 4, 8, 7, and 8 amino acids changed in the predicted protein of Qingyezhuma, Huazhu 5, Chuanzhu 8, and Dazhuhuangbaima, respectively, by comparing with the protein of QDY (Fig. S3). Analyzing the putative protein sequence among these four varieties and two parents, an identical amino acids sequence in MYB domain was observed in three thin-bark varieties (Qingyezhuma, Huazhu 5, and QDY). However, in two thick-bark varieties Dazhuhuangbaima and Chuanzhu 8, there is a change of amino acids (Tyr/Cys) found in the MYB domain (Fig. S3), which possibly have an important influence on the protein function. Therefore, in three thick-bark varieties, either a truncation or a change of amino acids in the MYB domain was observed in the putative protein. This results further indicated that *evm.model.scaffold7373.133_D1* is a likely candidate gene of *qBT4a*.

## Discussion

### First high-density genetic map of ramie

The construction of useful genetic linkage maps and identification of agronomic trait-related QTLs will facilitate future genetic and breeding studies in ramie. However, until now, only a single low-resolution genetic linkage map, based on SSR markers, has been available^2^, and the resulting low number of mapped QTLs and large intervals made it difficult to use these QTLs for cloning or breeding programs. Compared with SSR markers, SNPs are more abundant in plant genomes, and their frequencies in plants are one in every 100–300 bp^22^. Their ubiquity makes them valuable for genetic mapping, as well, especially for generating high-density linkage maps. The recent origin and development of next generation sequencing (NGS) technologies have provided a powerful and cost-effective tool for high-throughput sequence determination and SNP discovery, and as a result, large numbers of high-density linkage maps have been successfully developed for many organisms, including non-model species^23–28^. In the present study, we developed a SNP-based genetic linkage map using GBS technology. Although the total length of this map (1942.9 cM) was shorter than the previously reported SSR-based map (2265.1 cM), the SNP-based map included a far greater number of markers (n = 4338) than the previous SSR-based map (n = 142), thereby reducing the average genetic distance between neighboring markers from 17.1 to 0.66 cM. Accordingly, the high-quality SNP-based map had a greater resolution than the previous one and will serve as a unique tool for molecular-assisted breeding and genomic studies that will contribute to the improvement of ramie.

### QTL clusters for RSB traits and FY

There were only 6 RSB QTLs reported in previous study^2^. In this study, the RSB traits were systemically dissected, and a total of 15 QTLs identified. Of them, 6 QTLs constituted 3 clusters, i.e., *qBW2* and *qFY2* formed a cluster, *qBT4c* and *qFY4b* made up of another cluster, whereas *qBT4a* and *qRFB4b* built up another one. The presence of many QTL clusters indicated that these four traits had a close correlation. Among these three QTL clusters, the cluster constituted by *qBW2* and *qFY2* increased traits by QDY alleles, whereas the other two improved the phenotypes by ZZ1 alleles. Therefore, these QTL clusters can be easily applied in ramie improvement by marker-assisted selection.

### Identification of a qBT4a candidate gene

Sequence comparison of the *qBT4a* region of the two parents revealed a putative MYB-like gene (*evm.model.scaffold7373.133_D1*), the ZZ1 allele of which included a 760-bp insertion that causes premature termination. The textile fiber from ramie consists of bast cell walls, so the formation and thickening of secondary cellular walls are important for fiber biosynthesis. Furthermore, secondary wall biosynthesis is regulated by a transcriptional network that includes NAC and MYB master switches and their downstream transcription factors^29^. Indeed, at least 16 MYB transcription factors are reportedly involved in regulating secondary wall biosynthesis in *Arabidopsis* ^16^. Among them, *MYB46* and *MYB83* are regulated by the NAC protein and function as master switches that activate a battery of downstream transcription factors, including a large number of other MYB genes^30,31^. Therefore, MYB genes clearly play important roles in secondary wall biosynthesis, and because secondary wall development contributes to fiber biosynthesis in ramie stem bark, thereby improving stem bark thickness, and because of the large fragment-insertion identified in the MYB gene of the ZZ1 allele of the *qBT4a* region, we deduce that the MYB gene *evm.model.scaffold7373.133_D1* is a candidate gene for the *qBT4a* locus. We also compared its putative protein sequences in three thick-bark and three thin-bark varieties, and found that an identical amino acids sequence in MYB domain was observed in three thin-bark varieties, whereas in three thick-bark varieties, either a truncation or a change of amino acids in the MYB domain was observed, which further validate our deduction.

## Materials and Methods

### Experimental population and phenotypic measurements

An FAL population that consisted of 134 lines derived from two ramie varieties, QDY and ZZ1, was developed using a previously described strategy^2^. The 134 FALs and the two parents were planted on the experimental farm of the Institute of Bast Fiber Crops, Chinese Academy of Agricultural Sciences, Changsha, China, in 2014. Field experiments were performed using a randomized complete block design with two replicates. For each FAL, 10 cuttage seedlings were planted into a two-row plot, with a within-row distance of 70 cm between plants and a between-row distance of 45 cm. Boundary effect was eliminated by planting with two-row ramie in the area around the population. In June of 2015 and 2016, when more than two-thirds leaves had shattered, the ramie was harvested, and the traits of RSB were investigated. The bark was ripped from the stems and weighed, after which BT was measured using a vernier caliper and bark from the middle of the stems, and BW was calculated as the mean bark weight per stem. Fibers were harvested from stem bark of individual plants and dried, and FY was calculated as the mean weight of fiber per stem. Finally, the RFB was calculated for each FAL as the ratio of FY to BW. Additionally, four varieties (Chuanzhu 8, Dazhuhuangbaima, Huazhu 5, and Qingyezhuma) from the National Ramie Germplasm Resource Garden located in the Wangcheng experimental farm of the Institute of Bast Fiber Crops were used for investigating the BT trait in 2017.

### DNA and RNA extraction

Young leaf samples were individually collected from the 134 FALs and two parents for DNA extraction, and stem bark was collected from 40-day-old ZZ1 and QDY seedlings for RNA extraction. All the samples were immediately frozen in liquid nitrogen and preserved at −80 °C until extraction. The total genomic DNA and total RNA were extracted using a DNA extraction kit (Tiangen, Beijing, China) and E.Z.N.A. Plant RNA Kit (OMEGA Bio-Tek, USA), respectively, according to the manufacturers’ instructions.

### High-throughput genotyping of FAL population

Restriction enzyme selection for the GBS was performed by predicting fragment sizes that would result from various enzyme combinations, according to the previously described ZZ1 genome, and eventually, the combination of *Mse*I and *Ecor*I was selected for constructing the GBS libraries. GBS libraries were constructed for each line by incubating 0.1–1 μg genomic DNA at 37 °C with *Mse*I (New England Biolabs, i.e., NEB, Ipswich, MA, USA), T4 DNA ligase (NEB), ATP (NEB), and *Mse*I Y adapter N, which contained barcodes, and then heat-inactivating the reaction mixtures at 65 °C. *EcoR*I (NEB) was then added to the *Mse*I digests and incubated at 37 °C. Thereafter, the fragments of each sample were purified using Agencourt AMPure XP (Beckman) and subject to PCR amplification using Phusion Master Mix (NEB), universal primer, and index primer. The resulting PCR products of each sample were purified using Agencourt AMPure XP (Beckman), pooled, and visualized on a 2% agarose gel, and fragments of 400–450 bp (including indexes and adaptors) were excised from the gel and purified using a gel extraction kit (QIAGEN, Valencia, CA, USA). The purified fragments were cleaned further using Agencourt AMPure XP (Beckman) prior to sequencing. Finally, paired-end sequencing was performed on the selected tags by Novogene Bioinformatics Institute (Beijing, China) using an Illumina 2500 platform (Illumina, USA).

### Sequence data grouping

Sequence data from each FAL were sorted from the raw reads using their barcodes. To ensure that the reads were reliable, the raw reads were filtered using three stringent filtering criteria that removed: (1) reads with ≥10% unidentified nucleotides, (2) reads with >50% of bases having Phred quality scores of <5, and (3) reads with >10 nucleotides aligned to the adapter, allowing ≤10% mismatches. The resulting clean, filtered reads from each individual were aligned to the ramie genome (genome accession ID: NHTU00000000) using the Burrows-Wheeler Aligner (BWA) software^32^ (settings: mem –t − 4 –k 32 –MR), and the alignment files were converted to bam files using SAMtools^33^ (settings: –bS –t). If multiple read pairs had identical external coordinates, only the pair with the highest mapping quality was retained.

### SNP identification

SNP identification was performed for the two parents and 134 FALs using SAMtools^33^. Because the two parents were heterozygous, the polymorphic markers between them were classified into eight segregation patterns (ef × eg, nn × np, ab × cc, aa × bb, ab × cd, lm × ll, hk × hk, and cc × ab)^23,28^, according to the CP model in JoinMap 4.0^34^. The numbers of SNPs, transitions, and transversions were also counted, and then, a Perl script was used to filter out the SNPs with more than two genotypes, retaining only polymorphic markers with the ‘aa × bb’ segregation pattern.

### Linkage map construction

Markers that contained abnormal bases or exhibited significantly distorted segregation (P < 0.001) or non-integrity (missing data in > 30% progenies) were filtered out using JoinMap 4.0^32^. The regression algorithm, three times circulation sequence, and Kosambi mapping function were used for marker distance calculation^35^, and the linkage map was drawn using Perl SVG.

### QTL and heritability analysis

QTLs for FY and the three RSB traits in two environments were detected using composite interval mapping with Windows QTL CARTOGRAPHER2.5^36^. Window size was set at 10 cM, and forward stepwise regression was used to identify significant markers as cofactors. The experiment-wise LOD threshold significance level was determined by computing 1000 permutations (P < 0.05) using a permutation test program in MapQTL^37^. These permutations can account for non-normality in marker distribution and trait values. The heritability for four traits was estimated in the population according to the description of Liu *et al*.^2^.

### cDNA sequencing and analysis

The RNAs of the two parents were treated with DNase I (Fermentas, Canada) and subsequently used as templates to reverse-transcribe first-strand cDNAs using M-MuLV Reverse Transcriptase (Fermentas), according to the manufacturer’s instructions. For each sample, 2-μg total RNA was reverse-transcribed in 20 μL reactions. A standard PCR protocol was then performed to amplify *evm.model.scaffold7373.133_D1* cDNA from the two parents, using gene-specific primers (Table S6). For sequencing, 8 μL PCR product corresponding to each parent was digested using 5 U *Exo*I (NEB) and 0.13 U shrimp alkaline phosphatase (Fermentas) and sequenced using a 3730xl DNA Analyzer (ABI, USA). Sequence contigs were assembled using SEQUENCHER 4.1.2 (Gene Codes Co.), and the resulting ZZ1 and QDY *evm.model.scaffold7373.133_D1* cDNA sequences were submitted to GeneBank with the accession numbers MF100996 and MF100997, respectively. DNA and protein sequences were aligned using CLUSTAL × 2.1^38^, and the intron and exon were identified by comparing the cDNA with the corresponding genomic sequence (Supplementary File S1). The conserved protein-encoding domain was identified using the NCBI conserved domain database^39^.

## Electronic supplementary material

Supplementary materials
