# Supplementary material for: QTL analysis of four main stem bark traits using a GBS-SNP-based high-density genetic map in ramie
Source: Sci Rep. 2017 Oct 18;7:13458. doi: 10.1038/s41598-017-13762-w (PMC5647422; doi:10.1038/s41598-017-13762-w)
Supplement: Supplementary file 1 — Supplementary materials [file 41598_2017_13762_MOESM1_ESM.pdf]

QTL analysis of four main stem bark traits using a GBS-SNP-based high-density genetic map in ramie

Chan Liu[1,*], Siyuan Zhu[1,2, *], Shouwei Tang[1,2], Hongwu Wang[3], Xia Zheng[1,2], Xiaorong Chen[4], Qiuzhong Dai[1,2], Touming Liu[1,2, ✉]

1 Institute of Bast Fiber Crops, Chinese Academy of Agricultural Sciences, Changsha, China

2 Researching Center of Deren Husbandry, Hunan, China

3 Xianning Agriculture Academy of sciences, Hubei, China

4 Yichun Institute of Agricultural Sciences, Jiangxi, China

* These authors contributed equally to this work.

Corresponding authors:

Touming Liu: liutouming@caas.cn

Supplementary materials

Table S1 Summary of sequenced results in the FALs and two parents

Sample	Clean reads	Q20(%)	Q30(%)	Tag	Mapping reads	Mapping rate	Average depth	Total SNP
QDY	6,363,904	96.01	90.62	322,543	5,547,740	87.18	17.20	286,918
ZZ1	6,437,948	95.62	89.75	350,185	6,044,197	93.88	17.26	240,243
1	2,027,900	96.67	91.98	189,402	1,803,107	88.91	9.52	129,211
2	2,728,428	96.41	91.58	248,065	2,483,131	91.01	10.01	171,870
3	2,018,740	96.27	91.00	185,734	1,831,338	90.72	9.86	153,112
4	1,986,286	96.08	90.55	151,446	1,496,288	75.33	9.88	159,580
5	1,915,360	96.44	91.48	164,158	1,648,144	86.05	10.04	152,384
6	1,796,890	96.38	91.21	137,039	1,384,091	77.03	10.10	110,174
7	1,877,658	95.56	89.29	146,739	1,596,522	85.03	10.88	115,894
8	2,608,740	96.47	91.62	217,053	2,274,716	87.20	10.48	206,218
9	1,824,194	96.37	91.39	150,688	1,571,678	86.16	10.43	129,519
10	2,108,022	96.42	91.41	174,633	1,870,321	88.72	10.71	139,895
11	2,056,482	96.61	91.66	176,276	1,820,927	88.55	10.33	150,410
12	2,323,316	96.52	91.57	180,139	2,017,556	86.84	11.20	164,446
13	2,656,772	96.44	91.44	190,197	2,189,170	82.40	11.51	166,480
14	2,112,722	96.53	91.75	154,222	1,705,693	80.73	11.06	106,691
15	2,357,368	96.40	91.44	176,208	2,019,341	85.66	11.46	117,845
16	2,078,824	96.26	91.17	177,994	1,810,197	87.08	10.17	141,255
17	2,257,890	96.31	91.43	171,397	1,923,077	85.17	11.22	134,292
18	2,584,344	96.48	91.64	208,696	2,201,745	85.20	10.55	146,855
19	2,047,506	96.03	90.47	148,597	1,676,170	81.86	11.28	159,434
20	2,404,852	96.41	91.32	200,877	2,099,163	87.29	10.45	161,806
21	2,480,558	96.55	91.72	202,001	2,201,811	88.76	10.90	149,091
22	2,588,126	96.19	91.03	185,014	2,088,804	80.71	11.29	187,790
23	2,538,478	96.75	92.32	185,802	2,125,573	83.73	11.44	160,469
24	2,430,512	96.66	92.13	180,471	2,064,585	84.94	11.44	171,105
25	2,557,702	96.66	92.05	184,672	2,027,699	79.28	10.98	155,140
26	2,435,202	96.65	92.06	189,462	2,127,657	87.37	11.23	138,687
27	2,537,518	96.09	90.82	184,864	2,220,217	87.50	12.01	159,127
28	2,399,682	95.80	90.24	178,286	2,023,545	84.33	11.35	145,460
29	3,203,110	96.56	91.83	233,608	2,786,941	87.01	11.93	214,200
30	2,332,224	96.22	91.10	181,270	2,026,600	86.90	11.18	124,801

31	1,790,608	95.00	88.53	138,456	1,535,472	85.75	11.09	90,087
32	2,783,512	96.52	91.80	202,861	2,428,242	87.24	11.97	183,153
33	2,233,298	96.66	92.08	179,130	1,943,565	87.03	10.85	130,698
34	2,136,716	96.17	91.05	167,707	1,885,023	88.22	11.24	139,074
35	2,554,870	96.38	91.39	208,456	2,228,391	87.22	10.69	194,841
36	2,665,378	96.17	91.04	202,237	2,303,476	86.42	11.39	137,144
37	2,855,868	96.47	91.66	208,647	2,370,225	82.99	11.36	202,832
39	2,731,428	96.63	92.12	209,258	2,356,241	86.26	11.26	185,042
40	2,576,704	96.50	91.78	188,672	2,160,299	83.84	11.45	160,007
41	1,411,886	96.60	92.20	129,750	1,182,024	83.72	9.11	106,662
42	1,452,114	96.81	92.65	118,809	1,266,499	87.22	10.66	98,708
44	1,713,162	96.90	92.77	136,048	1,420,345	82.91	10.44	93,778
45	1,567,998	96.07	90.95	120,633	1,277,505	81.47	10.59	101,817
47	1,471,476	96.48	91.88	125,015	1,302,661	88.53	10.42	84,754
48	1,503,570	96.82	92.59	119,729	1,293,069	86.00	10.80	106,799
49	3,334,698	96.79	92.27	194,202	2,893,615	86.77	14.90	164,911
50	3,225,328	97.12	92.93	226,511	2,835,915	87.93	12.52	223,636
51	2,675,688	96.95	92.52	194,095	2,296,141	85.81	11.83	148,495
52	2,821,176	97.07	92.86	205,363	2,515,697	89.17	12.25	188,425
53	2,635,214	97.16	93.05	188,407	2,277,835	86.44	12.09	187,005
54	3,190,060	96.99	92.62	202,214	2,731,907	85.64	13.51	227,287
55	2,811,980	96.55	91.61	192,672	2,452,717	87.22	12.73	202,691
56	2,813,732	97.07	92.95	191,733	2,463,766	87.56	12.85	232,232
57	3,298,530	96.93	92.55	203,386	2,863,673	86.82	14.08	146,199
58	2,844,782	97.09	92.88	191,638	2,447,211	86.02	12.77	196,533
59	2,959,358	97.00	92.71	196,419	2,577,011	87.08	13.12	166,680
60	3,331,462	96.90	92.41	204,873	2,888,710	86.71	14.10	201,256
62	2,404,578	96.88	92.41	191,649	2,180,960	90.70	11.38	215,375
65	2,894,052	96.97	92.45	219,369	2,520,548	87.09	11.49	215,949
66	3,011,916	96.89	92.34	188,465	2,353,925	78.15	12.49	144,056
67	2,877,618	97.06	92.75	222,265	2,518,257	87.51	11.33	184,908
68	2,847,176	97.12	93.04	193,052	2,106,195	73.97	10.91	234,132
69	2,693,638	97.13	92.95	186,569	2,298,536	85.33	12.32	153,598
70	2,840,924	97.05	92.77	198,129	2,494,446	87.80	12.59	201,815
71	2,744,444	97.12	93.03	202,989	2,368,882	86.32	11.67	227,318
72	2,912,998	97.14	93.01	196,597	2,418,145	83.01	12.30	186,125
73	2,544,796	96.74	91.89	160,132	2,052,894	80.67	12.82	153,169

75	2,875,880	96.89	92.34	199,522	2,454,124	85.33	12.30	141,814
76	2,969,242	97.11	92.93	235,449	2,641,736	88.97	11.22	210,427
77	2,867,714	96.97	92.46	210,958	2,388,041	83.27	11.32	211,604
78	2,902,726	97.34	93.46	201,302	2,393,480	82.46	11.89	211,206
79	2,783,376	97.23	93.19	198,173	2,360,237	84.80	11.91	206,404
80	2,749,638	97.22	93.09	189,086	2,287,940	83.21	12.10	180,643
81	2,970,030	97.11	92.94	194,483	2,431,036	81.85	12.50	210,175
82	2,642,786	96.81	92.15	206,977	2,365,744	89.52	11.43	211,022
83	2,664,134	96.67	91.75	171,099	2,231,134	83.75	13.04	137,763
84	2,914,876	97.33	93.28	195,596	2,577,955	88.44	13.18	236,918
85	2,544,096	96.91	92.37	177,944	2,167,362	85.19	12.18	224,149
86	2,356,938	96.13	90.46	149,601	1,958,272	83.09	13.09	136,923
87	3,044,244	97.06	92.82	219,510	2,686,805	88.26	12.24	222,968
88	2,609,444	97.02	92.76	171,591	2,206,659	84.56	12.86	164,376
89	2,243,734	96.62	91.75	165,794	1,924,867	85.79	11.61	112,723
90	1,811,612	96.72	91.81	150,473	1,566,428	86.47	10.41	117,147
91	2,090,308	96.76	91.93	149,197	1,759,033	84.15	11.79	112,166
92	2,146,326	96.86	92.19	156,683	1,602,864	74.68	10.23	153,576
93	2,109,424	96.82	92.27	175,224	1,838,095	87.14	10.49	159,444
94	2,193,062	96.72	91.97	158,584	1,872,877	85.40	11.81	139,302
95	1,913,032	96.59	91.72	137,451	1,665,905	87.08	12.12	126,428
96	2,256,874	96.87	92.36	179,799	1,959,804	86.84	10.90	172,704
97	2,192,590	96.81	92.18	166,955	1,906,624	86.96	11.42	155,415
98	1,931,152	96.35	91.00	136,292	1,649,131	85.40	12.10	91,747
99	2,073,180	96.65	91.69	162,534	1,805,755	87.10	11.11	112,370
100	2,048,720	96.94	92.37	143,961	1,730,412	84.46	12.02	142,626
101	1,955,530	96.62	91.59	145,264	1,635,672	83.64	11.26	130,237
102	2,106,384	96.94	92.52	158,543	1,793,121	85.13	11.31	148,383
103	2,191,836	96.86	92.29	174,715	1,923,608	87.76	11.01	160,802
104	2,189,762	96.91	92.31	172,021	1,928,351	88.06	11.21	122,898
105	2,106,804	96.83	92.20	159,302	1,833,561	87.03	11.51	140,192
106	2,028,390	96.60	91.55	142,833	1,671,141	82.39	11.70	137,857
107	1,849,610	96.37	91.03	136,793	1,567,648	84.76	11.46	94,032
108	2,222,130	96.96	92.38	147,200	1,812,026	81.54	12.31	105,796
109	1,910,302	96.62	91.63	143,465	1,621,155	84.86	11.30	134,970
111	2,227,792	96.78	92.09	159,652	1,915,820	86.00	12.00	193,775
113	2,224,980	96.77	92.05	167,288	1,933,846	86.92	11.56	159,415

114	1,985,392	95.72	90.06	146,509	1,654,083	83.31	11.29	116,175
115	2,451,862	95.97	90.88	198,466	2,109,694	86.04	10.63	136,505
116	2,207,704	95.87	90.64	181,064	1,899,363	86.03	10.49	127,516
117	2,332,088	95.88	90.59	189,405	2,053,155	88.04	10.84	134,703
118	2,188,874	95.87	90.62	164,158	1,892,747	86.47	11.53	152,867
119	2,189,736	95.71	90.06	166,514	1,896,597	86.61	11.39	134,753
120	1,895,998	95.57	89.72	136,528	1,568,707	82.74	11.49	130,925
121	2,185,854	95.92	90.66	159,316	1,908,601	87.32	11.98	112,444
122	1,823,052	95.80	90.28	130,603	1,511,073	82.89	11.57	120,642
123	1,777,064	95.00	88.41	121,555	1,482,977	83.45	12.20	139,821
125	2,316,534	95.91	90.66	166,072	1,966,289	84.88	11.84	126,211
126	2,144,854	95.78	90.44	163,383	1,880,540	87.68	11.51	150,866
128	2,167,952	95.92	90.50	167,826	1,866,230	86.08	11.12	162,661
129	2,117,888	95.82	90.36	164,342	1,812,691	85.59	11.03	181,216
130	2,280,556	95.79	90.41	169,802	1,925,552	84.43	11.34	126,803
131	2,454,242	95.85	90.64	175,075	2,114,906	86.17	12.08	156,859
132	2,075,202	95.87	90.56	148,044	1,664,010	80.19	11.24	127,925
134	2,425,322	95.70	90.28	191,288	2,150,082	88.65	11.24	142,208
135	2,458,906	95.71	90.43	173,195	2,142,427	87.13	12.37	166,437
136	2,480,974	95.84	90.57	175,147	2,145,553	86.48	12.25	145,154
137	2,006,012	95.50	89.62	151,584	1,703,801	84.93	11.24	137,261
138	2,220,146	95.71	90.13	159,505	1,899,702	85.57	11.91	123,313
139	2,120,366	95.90	90.64	168,333	1,846,609	87.09	10.97	155,225
140	2,176,642	95.81	90.51	162,041	1,850,504	85.02	11.42	143,183
141	1,923,104	95.80	90.38	151,825	1,609,341	83.68	10.60	137,076
142	1,932,128	95.92	90.72	152,984	1,664,461	86.15	10.88	124,085
143	1,974,796	96.03	90.93	153,334	1,697,403	85.95	11.07	99,700
144	2,058,112	95.92	90.60	143,561	1,761,488	85.59	12.27	145,122
145	1,992,698	95.78	90.16	132,159	1,763,004	88.47	13.34	81,774
146	2,495,194	96.56	91.92	202,983	2,129,294	85.34	10.49	219,337
**Total**	**329,017,904**			**489,057**				
**Average**		**96.47**	**91.59**		**2,072,477**	**85.49**	**11.59**	**156,300**

Table S2 Summary of the high-density genetic map

Linkage group	Number of markers		Length (cM)	Average marker interval (cM)	Maximum interval (cM)	Number of interval with > 10 cM
	All SNPs	Binned SNPs				
LG 1	434	289	117.6	0.407	11.5	1
LG 2	387	255	122.2	0.479	4.3	0
LG 3	99	86	98.3	1.143	5.8	0
LG 4	462	302	213.6	0.707	25.2	3
LG 5	275	160	116.8	0.730	21.0	1
LG 6	339	211	140.7	0.667	7.9	0
LG 7	254	189	150.2	0.795	14.0	1
LG 8	265	196	148.7	0.759	6.5	0
LG 9	247	189	106.9	0.566	4.4	0
LG 10	211	161	108.2	0.672	6.0	0
LG 11	177	150	105.1	0.701	10.3	1
LG 12	322	244	132.9	0.545	9.9	0
LG 13	298	201	144.4	0.718	6.8	0
LG 14	568	332	237.3	0.715	6.4	0
All	4338	2965	1942.9	0.655	-	13

Table S3 The genes identified in the candidate 5.1 cM regions of *qBT4a*

**Scaffold**	**Gene ID**	**Annotation**
scaffold46555	XP_010086871.1_D9	-
scaffold46555	evm.model.scaffold44589.78_D1	Protein VASCULAR ASSOCIATED DEATH 1
scaffold46555	XP_010096878.1_D4	Uncharacterized protein
scaffold77073	XP_010090924.1_D3	Cytochrome b-c1 complex subunit Rieske-1
scaffold77073	XP_010109995.1_D105	Uncharacterized protein
scaffold77073	XP_010096980.1_D64	Uncharacterized protein
scaffold77073	XP_010105230.1_D157	Uncharacterized protein
scaffold77073	evm.model.scaffold41653.3_D1	30S ribosomal protein S2
scaffold77073	VIT_03s0132g00110.t01_D205	Uncharacterized protein
scaffold459380	evm.model.scaffold15095.22_D1	
scaffold460912	XP_010106767.1_D29	Putative ribonuclease H protein
scaffold461852	XP_010106767.1_D9	Non-LTR retroelement reverse transcriptase-like protein
scaffold92967	XP_010091047.1_D1	Probable alpha-mannosidase I MNS5
scaffold92967	XP_010091049.1_D1	Calmodulin-binding protein 60 A
scaffold92967	VIT_05s0062g00060.t01_D12	Retrovirus-related Pol polyprotein from transposon TNT 1-94
scaffold92967	XP_010091051.1_D2	UDP-glycosyltransferase
scaffold92967	XP_010111544.1_D1	LIM domain-containing protein WLIM1
scaffold92967	XP_010091042.1_D2	Uncharacterized RING finger protein C2F3.16、
scaffold92967	evm.model.scaffold40715.61_D1	LOB domain-containing protein 27
scaffold92967	XP_010106306.1_D2	Protein TRANSPARENT TESTA 12
scaffold92967	XP_010108321.1_D1	Uncharacterized protein
scaffold92967	XP_010091046.1_D1	Peroxidase 65
scaffold92967	XP_010111542.1_D1	Uncharacterized protein
scaffold92967	evm.model.scaffold17401.38_D1	Succinate dehydrogenase subunit 7B
scaffold92967	XP_010091045.1_D1	Putative DNA glycosylase At3g47830
scaffold92967	XP_010111543.1_D1	Pentatricopeptide repeat-containing protein At5g66520
scaffold92967	XP_010091044.1_D2	U-box domain-containing protein 38
scaffold92967	XP_010091052.1_D1	Uncharacterized protein At5g50100
scaffold116363	XP_010091790.1_D1	Acid phosphatase 1
scaffold116363	XP_010091822.1_D1	NADH dehydrogenase [ubiquinone] 1 alpha subcomplex subunit 9
scaffold116363	XP_010091838.1_D1	Protein QUIRKY
scaffold116363	evm.model.scaffold30633.17_D1	Uncharacterized protein
scaffold116363	XP_010091823.1_D1	ABC transporter C family member 5
scaffold116363	evm.model.scaffold7373.108_D1	Putative F-box/FBD/LRR-repeat protein At4g13965
scaffold116363	XP_010091820.1_D1	Galactosyltransferase
scaffold116363	XP_010091794.1_D1	Glutathionyl-hydroquinone reductase YqjG
scaffold116363	XP_010093087.1_D1	Protein JASON OS=Arabidopsis thaliana
scaffold116363	XP_010091798.1_D1	Mitochondrial import receptor subunit TOM9-2
scaffold116363	XP_010091826.1_D1	WD repeat-containing protein 70
scaffold116363	XP_010091805.1_D1	Auxin-responsive protein IAA11
scaffold116363	XP_010091825.1_D1	Tyrosine decarboxylase 1

scaffold116363	XP_010091802.1_D1	Uncharacterized protein
scaffold116363	XP_010091791.1_D1	Uncharacterized protein
scaffold116363	XP_010091837.1_D1	Sulfofructose kinase
scaffold116363	XP_010091833.1_D1	WD repeat-containing protein 26
scaffold116363	XP_010091799.1_D1	Uncharacterized protein
scaffold116363	XP_010091831.1_D1	RmlC-like cupins superfamily protein
scaffold116363	XP_010093083.1_D1	Exocyst complex component EXO84B
scaffold116363	XP_010091818.1_D1	Photosystem II reaction center PSB28 protein
scaffold116363	VIT_07s0005g04110.t01_D1	Cellulose synthase A catalytic subunit 4 [UDP-forming]
scaffold116363	VIT_16s0148g00020.t01_D1	Probable LRR receptor-like serine/threonine-protein kinase
scaffold116363	VIT_03s0063g01600.t01_D5	-
scaffold116363	evm.model.scaffold7373.144_D1	Pre-mRNA-processing factor 39
scaffold116363	XP_010091821.1_D1	-
scaffold116363	XP_010093085.1_D1	Protein NRT1/ PTR FAMILY
scaffold116363	XP_010093085.1_D2	Protein NRT1/ PTR FAMILY
scaffold116363	XP_010091806.1_D1	Leucine-rich repeat receptor-like protein kinase PXL2
scaffold116363	XP_010091830.1_D1	Alcohol dehydrogenase class-3
scaffold116363	XP_010091797.1_D1	Cysteine-rich repeat secretory protein 56
scaffold116363	XP_010091795.1_D1	Histone-lysine N-methyltransferase SUVR2
scaffold116363	XP_010093086.1_D1	Heterogeneous nuclear ribonucleoprotein 1
scaffold116363	XP_010091788.1_D1	Probable LRR receptor-like serine/threonine-protein kinase
scaffold116363	XP_010091804.1_D1	W9RB84_9ROSA Uncharacterized protein
scaffold116363	XP_010091841.1_D1	BRCA1-associated RING domain protein 1
scaffold116363	XP_010091801.1_D1	Putative G3BP-like protein
scaffold116363	XP_010091832.1_D1	Activating molecule in BECN1-regulated autophagy protein 1
scaffold116363	evm.model.scaffold7373.177_D1	Receptor-like serine/threonine-protein kinase ALE2
scaffold116363	VIT_07s0005g04590.t01_D1	Tetratricopeptide repeat protein 4 homolog
scaffold116363	XP_010091819.1_D1	Subtilisin-like protease SBT1.2
scaffold116363	XP_010091827.1_D1	DENN domain-containing protein 5B
scaffold116363	evm.model.scaffold7373.133_D1	MYB domain protein
scaffold116363	evm.model.scaffold29199.37_D2	60S ribosomal protein L23
scaffold116363	VIT_07s0005g03910.t01_D1	Phospholipid--sterol O-acyltransferase
scaffold116363	XP_010091803.1_D1	W9QPL3_9ROSA Uncharacterized protein
scaffold94597	XP_010107729.1_D52	Putative AC transposase OS=Zea mays PE=2 SV=2
scaffold77675	XP_010097746.1_D2	Protein ECERIFERUM 1
scaffold104223	XP_010109995.1_D24	Uncharacterized protein
scaffold104223	VIT_07s0005g05830.t01_D6	-
scaffold104223	XP_010087579.1_D22	Uncharacterized protein
scaffold111657	evm.model.scaffold38181.17_D4	Glycolipid transfer protein 3
scaffold111657	XP_010097326.1_D29	Retrovirus-related Pol polyprotein from transposon TNT 1-94
scaffold50309	XP_010102096.1_D1	Uncharacterized protein
scaffold50309	XP_010088789.1_D29	Uncharacterized protein
scaffold50309	XP_010096916.1_D5	Ty3/gypsy retrotransposon protein
scaffold50309	XP_010105983.1_D73	Uncharacterized protein

scaffold50309	XP_010103526.1_D26	Uncharacterized protein
scaffold50309	XP_010087321.1_D64	-
scaffold34965	XP_010097762.1_D1	Acyl carrier protein 1
scaffold34965	VIT_03s0110g00150.t01_D15	Putative uncharacterized protein
scaffold34965	XP_010096171.1_D12	-
scaffold34965	XP_010097763.1_D1	Putative invertase inhibitor
scaffold34965	XP_010096172.1_D11	Uncharacterized protein
scaffold34965	ATMG00750.1_D56	Uncharacterized mitochondrial protein
scaffold109281	VIT_00s0208g00040.t01_D6	Putative uncharacterized protein
scaffold109281	XP_010097744.1_D29	-
scaffold109281	XP_010091858.1_D2	Uncharacterized protein
scaffold109281	XP_010102941.1_D16	Uncharacterized protein
scaffold109281	XP_010087322.1_D3	Uncharacterized protein
scaffold109281	XP_010098788.1_D94	HAT family dimerisation domain containing protein
scaffold109281	XP_010102094.1_D3	Protein MKS1
scaffold48691	XP_010100479.1_D3	Copia protein
scaffold48691	XP_010087170.1_D4	Uncharacterized protein
scaffold48691	VIT_18s0001g15710.t01_D1	Peroxiredoxin Q
scaffold48691	XP_010108667.1_D1	LRR receptor-like serine/threonine-protein kinase RCH1
scaffold48691	VIT_00s0227g00030.t01_D17	Mutant gag-pol polyprotein
scaffold48691	XP_010087406.1_D2	Uncharacterized protein

Table S4 The results of sequence comparison for four genes in two parents

Gene ID	cDNA in ZZ1	cDNA in QDY	SNP	Numbers of difference AA
VIT_07s0005g04110.t01_D1	CL2178Contig1	comp48284_c1	29	3
XP_010091051.1_D2	Unigene7237	comp42331_c0	13	3
XP_010091820.1_D1	CL14335Contig1	comp47252_c0	9	4
evm.model.scaffold7373.133_D1	T2_Unigene_BMK.20714	comp45758_c0	14	5

Table S5 The EST ID of evm.model.scaffold7373.133_D1 in 6 varieties investigated

Variety	Trait value of BT (MM)	EST ID in transcriptome
ZZ1	0.94	T2_Unigene_BMK.20714
Dazhuhuangbaima	1.00	comp36503_c0
Chuanzhu 8	1.03	comp38697_c0
Qingyezhuma	0.63	T1_Unigene_BMK.21700
Huazhu 5	0.86	comp83814_c0_seq1_39
QDY	0.72	comp45758_c0

Table S6 The primer for amplify the cDNA of evm.model.scaffold7373.133_D1

Name	Forward Primer	Reverse Primer
MYB-1	TGCCCTCTCTCGATTCCCAT	GGCTTCCATGAGTGAGAGGA
MYB-2	GACAACAAGCAAAGGGTGGT	GCAAGTGGCTTTTGACATGA
MYB-3	GTTCAAAAACCGCCTTCAGA	CCGTTGTTGTTTTTCGAGGT
MYB-4	GCTGCATGAACAGCTTGAGA	TCTTTAAGCAACGTCGTCCA

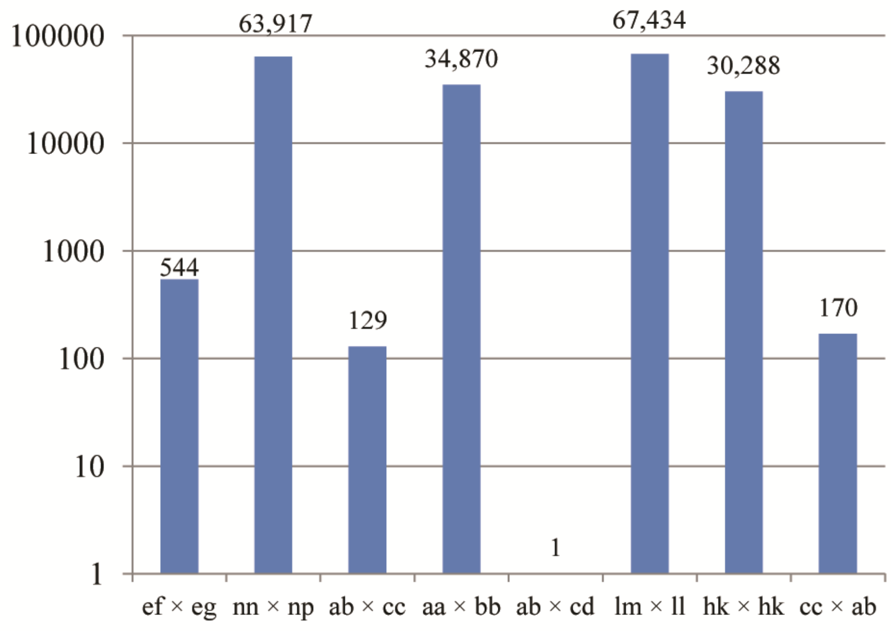

Fig. S1 The eight segregation patterns of SNPs and their number identified in this study

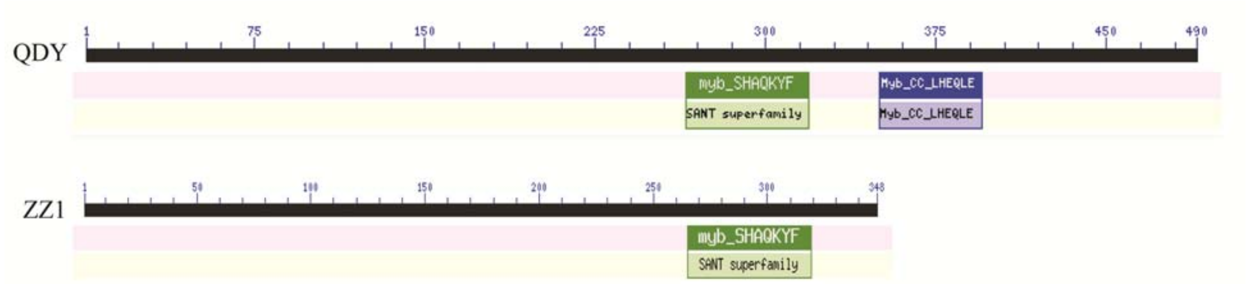

Fig. S2 The conserved domain of evm.model.scaffold7373.133_D1 protein predicted in CCD database of NCBI. A absence of a part of MYB domain in ZZ1 allele was observed.

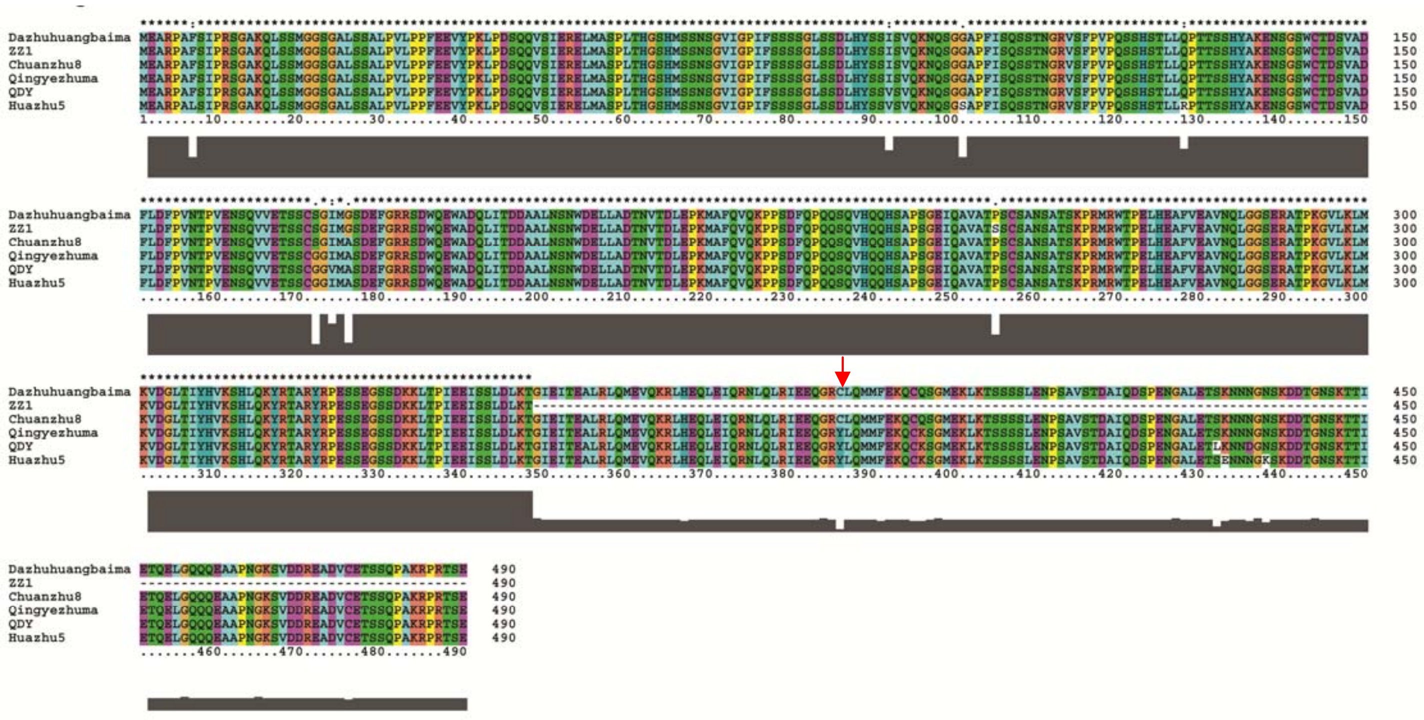

Fig. S3 Clustal X alignment of *evm.model.scaffold7373.133_D1 encoding* protein sequences in six varieties. The red arrow indicates a substitution of amino acid in the MYB domain.

File S1 The sequence of evm.model.scaffold7373.133_D1 in ZZ1 genome

> Genome sequence

CAAAAAGTTTTCCTAAAAAAAGAAGTTAAGATAATTTCAATAAAATTAAAAATACACGTTATTTAAAAAAAGTTAATAAAATA
TTGGAAGTTTTTTTTTGTTGTTGTGACCTGTGCGAAGTATTCTGACATATTAAATTAGAATTTTGCAAAAAAAGAAAAAAAAA
ATCAAACTAGAAGCCAAAGCCCCACGTTGCGAGAGGCATCAGATATTACCTCCAACTTCACCAATCACACGCACTCACATCTC
ACCCCGTGGACCCCACAACAAATGCCTCCACGCGCTCACCCTTAAAACGACACATTCGGGTACGTTTATTCCACGCGCTCCAG
TGGCGCGTGGAAGAGCATAGACTGTTTCCTTATGTCCCGAAGAAAGACCAAAGAGAGAGAGAAAAAAGAAAAGAAAAGAAAAG
AAAAAAAAAAAAACAGAGAATGGCAAAAGCAAAGCGCACTCGTCACCGTTCAATTCCTCGCCGGTGAGAGAATTTCCTTTTTT
TTTTTTTTCTAATAAATAATAATAAAAAAATGAAAAAAAATACCGGGCTGTCACCGTAGCTTTACCTCTCTTTTCCTCTTCCC
GTTCGACCGAATTTCTGGTCTCGATTCGATTCTCGATCTCTCTCTCTCTCTCTCTCTGCCCTCTCTCGATTCCCATTTTTT
TTTTTCTTTTAAACTTTTCTCTATCAGTTTTCGGGTAAGATTTGGGTCTGTAAGTTTCGAAAGTTTCGTGCTTTTAAGCTATT
TGGCGGTGAAATTCTCTGCCTCTGTTGCTCTTTTAGCGTGATTTTTTTTTCCCCTTATACTGTTTCATGGCGGATAATGTGTT
GCATATTTCTTATTATTATCTTTTCATGCACTTAATTGGTTTAGTTTCGATAAACGAGATTCGTTTTGAGCATGGTGTGACAT
AAGCCAGTTTCTATGCAAATTTTTTCGGTGGATCAAATGTTTTTGATGATAGGACTGTTCTCGGAGTTTGTTGTTAGCTTCAA
GTTATGATTTTTATCAATTCTCGGAATATACTTATAAATTTCTCTCTTACCTTAGCTGAGAAATTGGCTAAAGTTTTGTTTTT
GTTTTTTCGTTTTTTTGTTTTTATCAGTTCTCAATCAGAGAGTATTATTCTAGAAATTGTATTAGACAGACTATAGGACTGAT
TAGAGATTTCCTGATATGTGTCATTTATGTCGAATCTCACGATTGTTGTTCGTCGTCCTAGACAATGATATTCATCGGTTGAG
AAATAAAAAACTAATGAAAGAAAAAACAAAATGAGGGCTTGATTTGTGTAAGCATGTATTTGATGATTGTTCACAGATTTCCA
TATTTTATTTCTTTGCCATTAATGGTACTTCATTTTCCTTTCATTTCCCAGAGGTGGTGATTGATGACCCTTTATTTGTTTTG
GATATTGACAACAAGCAAAGGGTGGTTTTACTTATAGTAGGTCTTGAGTTGGTGAGCTTTATTCTTACAGCATCTTGATCACA
TACAAATACATAAATATACACATATATAATATGTGATCCATATTGTAATTCACTAATAGTTGTTTTGGTGTTAGGAAGACAAT
GGAGGCACGCCCTGCCTTTTCAATTCCGAGGTCCGGCGCGAAGCAACTCAGCAGCATGGGCGGTTCCGGGGCGCTCTCTTCGG
CTTTGCCGGTGCTTCCTCCGTTTGAAGAGGTATATCCAAAGTTACCGGACTCTCAACAAGTTTCCATTGAAAGGGAACTAATG
GCAAGTCCTCTCACTCATGGAAGCCACATGAGCTCCAACTCTGGAGTTATTGGTCCTATCTTTTCGTCGTCTTCGGGACTCTC
ATCAGATCTCCACTACTCGTCCATTTCGGTTCAGAAGAATCAGTCGGGCGGCGCTCCTTTTATATCTCAGTCGTCGACTAATG
GGAGGGTATCTTTCCCCGTACCGCAATCTTCGCATTCGACACTGCTTCAGCCTACAACATCGAGTCATTATGCCAAAGAAAAC
AGTGGCTCCTGGTGTACAGATTCCGTTGCGGATTTCCTTGATTTTCCTGTTAATACCCCTGTTGAGAATAGTCAAGTAGTAGA
AACCAGTAGTTGTAGCGGCATCATGGGGTCCGACGAATTTGGGAGGCGTAGCGATTGGCAAGAATGGGCTGACCAGCTCATCA
CTGACGATGCTGCTTTAAATTCTAACTGGGACGAACTTCTCGCCGACACCAACGTAACGGATTTGGAACCAAAGGTTGGATCC
TCTTATATGTATGGCGCTTAAGATGCTTTTGCAAAGTTCCTAAAAATAAATACATTTGCATAGTGAAGTTTGGAATTTTGGTG
TGTAATTAGTCAACAAGCATTTTGGCTTCTCGTTTTGTTGCTTCAGATGGCGTTCCAGGTTCAAAAACCGCCTTCAGATTTTC
AACCTCAGCAGTCCCAGGTTCATCAACAGCATTCTGCTCCATCTGGAGAAATCCAAGCCGTTGCTACTCCGTCTTGTTCGGCT
AACAGTGCCACATCCAAGCCGCGCATGCGATGGACACCCGAACTGCACGAGGCCTTTGTGGAGGCTGTTAACCAACTTGGTGG
CAGTGAAAGTGTGTATCTCTTTGGCTGCTTGGTTCTTAAATATTATAAGCTTCTGTTTCAGTTGGGTTAATTTTTATTTATTT
ATTTACTTATTCTTATTTTTAACTTTTCAGGGGCAACACCTAAGGGCGTCTTGAAACTTATGAAGGTTGACGGCTTGACTATC
TATCATGTCAAAAGCCACTTGCAGGTATTATAATCTTTTGGCATTAGATCGTTTCCTACAAAATGCATTGCTAGTGTGTTTGT
GTTTTTCTGTGAGCTTTTATCTAATCCTTGAGCTTAATTCGTGAATTGCAGAAATATAGGACAGCTAGATATAGACCGGAGTC
TTCAGAAGGTATGTAGCAAAAATCTCTTAAGTCCTATTTTTGCATACAATCTGGTTGCTTTCTTTTTTATCTGTATTGGCTAT
TCCCTCCTTATTTTGCTGCATTAGTCTCTTGGTGCTTGCACTTTTCTCTCTTTCTACTAGAAACAGAAAGTGGCAGGTATGAC
ATGTATGCGCTTTTTTCCTGCATTCCCGCTGTGGAGGTCGGGAAGTTTTTGGTTCAACTACTGCTGCTATTCACCTTTCCCCT
CCCCCCCACAATATATGATTAATCACCATTGCGTTAACTGGTTTTACTTTCTATTGCGTATACATGTAAAGATCTAACATCAA
CTTGATGTACTGAATTGTACTCTCTGTGTGCAGGATCCTCAGATAAGAAATTGACTCCCATTGAAGAAATATCATCTCTTGAC
TTGAAAACGTGAGTCAATCTGTTAGCAATACTTAAAAATATGAATTTTTTTTTTTCGTTGATTATTTGGCTTGTGCAGTTTT
TAACTTTTTATGATTATCTTGTATACGTCTTAGCAATTTTTAGTGACGTTTAACTGGACACCGTTTGTCCTTCCAAATTGATT

GTGGTCGTAACCTATATGCTTGAAACATTGATGGACATGCTATAAGCTCTAGTAATGTAGTCTGGGGATGTCTTGAGCTTGAA
AACTCCATCTATGTCTAGAAAATAGGACATGAATATAATCTAGTTTCCAAGGTTTCTTGCCGGTTTGTAGTTTAGAAGGAAGC
CAGGATGAACAGAGGCTGCTTTTTAGCTCCTTCTGCAACTTTACCAGTCACTGGGGTTCTTAATTTGGATATATCCTTGAGCT
GATGAACCTGCTGTGGACTCTCAAGTCGCTCTTTGTTGCAGTGTGTTGCCAAGTACATGGCTTTTAACCAAATAACCCAAGTG
ATGGCAAAATTAATATGTAAGACAGCAAAGTAAAATCTGCTCAAAGTAAAGAAGTATTTTAAGCTAGATAGGGGTACATAAGC
TGTTTACAGTGGTCTCTGCTTAAAGGTGGAAAGCTACCGTTCTGTATTTCAATCAATGAGTGGAGGCGGTGATCTAGAAGGGG
GCCCATTGTCACATTGTTCTACCTCTAAATATGGTTACGTTTTTCGTTTCAATATCTCATTCATTAGGTTGAATTATACTGAA
TATATTCTACTTGCTGAACAGGGGTATTGAAATTACTGAAGCTCTGCGTTTGCAAATGGAAGTCCAGAAGCGGCTGCATGAAC
AGCTTGAGGTCTGACTCTTCACCATGGGAATTCATTGCTTTATATAATATTGATTTCACGATCATACTAAAAAAATGGGCCAA
TGGTTTTGTCCACCTTGCAGAATTTCTTGGGCTAAATCCCAGTGCCTTTAAATGGTTTTTTCTGTTCTTTTTGATGTCCTATT
GAAATGTTTCTCGCTTCTGCAGATACAACGAAACCTGCAGTTGAGAATAGAAGAACAAGGCAGGTGCTTGCAGATGATGTTCG
AGAAGCAATGCCAATCGGGTATGGAAAAGCTGAAGACTTCATCCTCCTCTTTGGAGAATCCCTCTGCCGTGTCTACTGATGCA
ATTCAAGATTCCCCTGAAAATGGTGCATTAGAAACCTCGAAAAACAACAACGGGAACAGCAAAGACGATACAGGCAATTCAAA
AACCACAATAGAAACCCAAGAGCTCGGCCAGCAGCAAGAGGCTGCACCCAACGGGAAATCGGTAGATGATCGCGAGGCAGACG
TATGTGAGACAAGCTCCCAACCCGCCAAGCGCCCGAGAACAAGTGAATAAGCCGTACCATCGATCAACTTAGCATTAGCGTCA
AACTAACAGTTCACTAGTACATTATTAATCTTGACTAAGGCAGGTGTTGCGCTGTAGATTGAAAGAGCTTCGAAGAATTTGAG
GTCCTTCTCTCGTGTTCTAAGTGTGGTTTTTATTATCATGAGCTCACTTGATGCAGTCTCTCTATCTGGATGATGATTTGGAG
CTCGTTACAAGTTTTGTTCCCTTTATTATTCTTAAACCTCTACTAGTACACTCTTCTTGCCAAAGTAAGGATGAGGCTCTCAT
AATAGATTGTGCCTTTAGAGGTGTTTGTATCTTTGAGGGACTTGAATGTTCTCTAGTTTTAAGCTATGGATAATAAATGTTAC
TGCCCAATAATGCTAATAACTTCCTAATCTGAAGATTAGCACTGTACATTCGGTTTCGCACTCCGGTTCAGACCGGTGATCGG
TACTTGGGTAGCAATGACTGTTTCAGACCTTTTTTTCTTCTTGAAGTTTCAAGCTTGTGCTTTTCTACTTATGCTAACTGTGT
GCGTTAGATGGACGACGTTGCTTAAAGATCTAAAACTCCATCTTACCGAAAATACATACGATCAGCCAACCGAACTTTGAGCT
TAAAGAATAAAGCTTATGCCAAGAAAGTAAAACATGAAACATCAGACATTGACAAACTGACAAAAGATTGTTCCACAAATGTG
TTTTGAGCGAGTTTATTATTGATCGTTGAAGGCTGTACAATGAAGCTTGAAACAATGGAGGGAGCAAAATCAAACATAGATGA
TAATCCAAGCGATTCATGTAGACCTCGCAAACTGACAGTTTCACATTCTTTATTCCTTTTATCATATTTTTCAGAGAAATTGA
TTAATTTGGAGGGCAAAAAAAAAAAAAAAAAGAGACAACTGATGAATTTTGGTTATGAAGAAAAGTGGGGTACTTGACATATA
GCAAACAAGACGGTGCCAAAAAGAAGTGATTTTATTGTTTGTTTAGGAAAAAACTCATCTACCCTTGGCCTCATAGACGCCTT
TGAACCTAAGCAACCAAAAAAAAAAAAAAAAAAGACAGACCAAACCCATCTTCTCTTGAACCAAATTCAATCGCTCCTCTCAT
CACTAACGTCTCTTATAATCAAACTAATGACTTCCCAACTCACAGTAGGTAGACTCAACTCACTTACACCTCACACAATGTTC
CTTTTTTTCTTTCACTTTCACCTTCAAAACCTTCAATATAACTATACCTTCATTTCCTCTTCTTCCTTCACAACCCTTTCGCA
